# A Mechanism for Ovulation Number Control

**DOI:** 10.3389/fendo.2022.816967

**Published:** 2022-07-14

**Authors:** Michal Shilo, Avi Mayo, Uri Alon

**Affiliations:** Department of Molecular Cell Biology, Weizmann Institute of Science, Rehovot, Israel

**Keywords:** mathematical model for ovulation, ovulation number control, polycystic ovary syndrome, biphasic control, biphasic control of androgen, hyperanderogenism, mathematical model for follicular growth, Lacker’s model

## Abstract

Every menstrual cycle, many follicles begin to develop but only a specific number ovulate. This ovulation number determines how many offspring are produced per litter, and differs between species. The physiological mechanism that controls ovulation number is unknown; a class of mathematical models can explain it, but these models have no physiological basis. Here, we suggest a physiological mechanism for ovulation number control, which enables selection of a specific number of follicles out of many, and analyze it in a mathematical model of follicular growth. The mechanism is based on a signal, intra-follicular androgen concentration, that measures follicle size relative to the other follicles. This signal has a biphasic effect, suppressing follicles that are too large or too small compared to others. The ovulation number is determined by the androgen inhibitory thresholds. The model has a scaling symmetry that explains why the dominant follicles grow linearly with time, as observed in human ultrasound data. This approach also explains how chronic hyperandrogenism disrupts ovulation in polycystic ovary syndrome (PCOS), a leading cause of infertility. We propose specific experiments for testing the proposed mechanism.

**Graphical Abstract:**
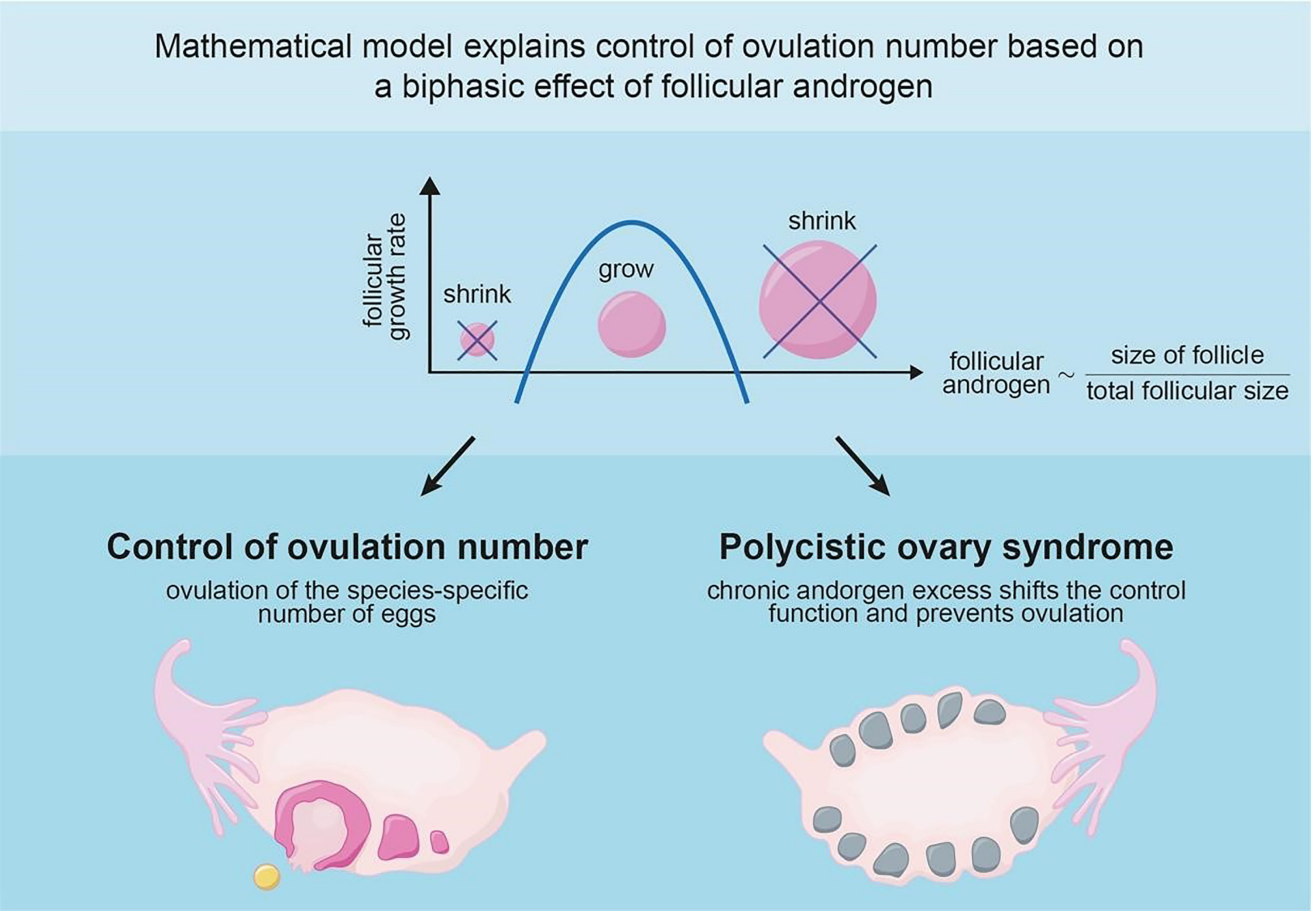


## Introduction

**Figure 1:**
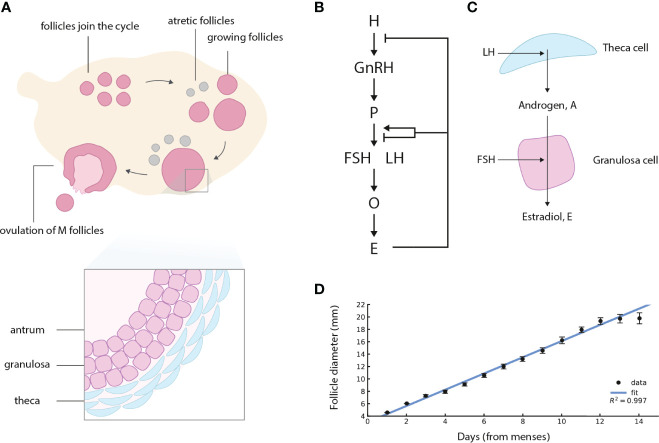
Ovarian follicles compete for ovulation during the follicular phase of the menstrual cycle, under control of the Hypothalamus-Pituitary-Ovary (HPO) axis. **(A)** Follicles join the menstrual cycle and grow, *M* of them “win the race” and ovulate, and the rest die (atretic follicles). In this schematic figure, *M* = 1 is shown. **(B)** The HPO axis controls circulating hormone levels. H denotes hypothalamus, P pituitary, O ovaries (specifically the ovarian follicles), and E estradiol. Estradiol control of gonadotropin production changes sign from negative to positive at high sustained estradiol levels. **(C)** Follicles in the ovary produce steroid hormones. LH induces theca cells to convert cholesterol to androgen. Most of the androgen goes to the circulation, and a small amount is converted to estrogen by the granulosa cells under control of FSH. **(D)** The dominant human follicle size measured by ultrasound grows with an approximately constant velocity (linear growth) in the follicular phase, adapted from (1). Regression line for the first 13 days of the follicular phase is shown, after which the control of Estradiol changes sign.

In mammals a large number of follicles start growing every menstrual cycle, but only *M* of the follicles ovulate, and the rest die in a process called atresia (**Figure 1A**) (2–6). The ovulation number *M* is species-specific. In humans and elephants *M* = 1 except for rare twin events. In young mice *M* ≈ 8 (7). The question of how *M* follicles are chosen is called the *“choose M”* problem (8).

The fitness of an organism depends on its ovulation number *M*. The higher *M* the more offspring, but *M* that is too high places a load on parental care, leaving fewer surviving offspring. The optimal *M* is at intermediate values, an observation known as Lack’s principle (9, 10). It is not clear how the ovulation number problem is solved physiologically: what determines *M*?

Ovulation is regulated by the endocrine Hypothalamus-Pituitary-Ovary (HPO) axis (**Figure 1B**) (11–14). In the HPO axis, the hypothalamus secretes gonadotropin-releasing hormone (GnRH), which promotes secretion of two gonadotropins from the pituitary, follicle stimulating hormone (FSH), and luteinizing hormone (LH). LH promotes production of androgen (A) in theca cells of the follicle. Androgen is converted to estradiol (E) and other estrogens by the granulosa cells of the follicle under control of FSH (**Figure 1C**). FSH also promotes follicular growth and survival.

The ovarian follicles implement a negative feedback loop, in which estradiol inhibits the production of upstream hormones in the HPO axis. At late stages of the follicular phase, the phase of the menstrual cycle in which follicles compete and in which *M* are chosen to ovulate, the feedback switches sign and becomes positive. High estradiol activates LH production, triggering the LH surge which causes ovulation. Ovulation is dysregulated in a prevalent disorder called polycystic ovaries syndrome (PCOS), linked with excessive androgen levels and impaired fertility (15–18). In cases of anovulatory PCOS, competing follicles stop growing prematurely and regress (17).

The mechanism that determines the ovulation number remains a mystery, and is a topic of current research (5, 6, 19, 20). The ovulation number is known to be regulated by circulating factors, since removing one ovary does not reduce the total number of eggs released during ovulation by half (7, 21, 22). An elegant theory of the ovulation number was developed by Lacker et al. (4, 23–25) in the 1980’s. Lacker’s model can provide a choice of specific *M*. In the model, circulating estradiol secreted by the follicles provides systemic control over ovulation number, with a biphasic effect in which follicles that are too small or too large are eliminated. The biphasic effect is central to the model, but, as acknowledged by Lacker, it lacks a physiological mechanism for this effect. Defining a physiological mechanism can advance our understanding, and offer experimental tests and therapeutic points of intervention.

Lacker’s model is also inconsistent with the dynamics of follicle growth. It shows super-exponential growth of the dominant follicles, reaching infinite size at a finite time. This is in contrast to more recent measurements of follicular growth profiles in women, not available at the time the model was formulated, that show approximately linear growth with time of the dominant follicle (**Figures 1D** and **S1**) (1, 26). Lacker’s model and later variants (27, 28) are also not consistent with follicle dynamics in PCOS (17), because they show follicles that are growth arrested and persist at an intermediate size, rather than follicles that grow and then shrink.

It would be important to develop a model for ovulation number control based on physiological mechanisms, which can explain the ‘choose *M*′ problem, linear follicle growth and the origin of conditions such as PCOS.

In this paper, we combine multiple lines of evidence to propose a physiological mechanism for ovulation number control, and to develop a minimal mathematical model for follicular growth. The main regulators of growth in the model are systemic FSH that enhances follicle growth, and local androgen in each follicle which has a biphasic effect on follicle growth. The model explains linear follicle growth based on an invariance property. It provides a mechanism for how high androgen levels cause PCOS and its anovulatory dynamics, and a framework to understand the frequency of dizygotic twin ovulations.

## Results

### Biphasic Model for Ovulation Control Based on Local Androgen

We sought a mechanism by which follicles that are too large or too small compared to the other follicles can be removed. Such a principle was assumed in Lacker’s mathematical model, but with no physiological underpinning. We suggest a candidate for such a biphasic control based on the local androgen concentration in each follicle, as we describe next.

Androgens are produced by follicles in the ovary and, to a lesser extent, by the adrenal glands. The androgens androstenedione and testosterone are produced in theca cells of the follicles. Small amounts of these androgens are used as the precursors for estradiol production by the adjacent granulosa cells of the follicle (**Figure 1C**). In sheep, humans and primates the main precursor for estradiol is androstenedione, whereas in rodents the main precursor is testosterone (29). Most of the androgens are released to the circulation, where they are diluted to low levels, while the androgen inside each follicle is at a much higher concentration.

Androgen is involved in follicular growth, as evident both by controlled experiments and disease states. The androgen receptor is expressed in granulosa cells (30). At low to physiological levels, androgen stimulates follicle growth and survival *in vitro* and *in vivo* (30). However, at high levels, androgen inhibits or interferes with follicular growth (31). This is seen in states with high androgen, such as polycystic ovary syndrome (PCOS), congenital adrenal hyperplasia (16, 32), hyperandrogenism in female-to-male transsexuals (33, 34), and aromatase deficiency (35). These states are related to a polycystic ovary morphology, in which ovarian follicles stop growing prematurely and ovulation is prevented (16). Excess external androgen can also induce PCOS symptoms in animal models (36).

**Figure 2:**
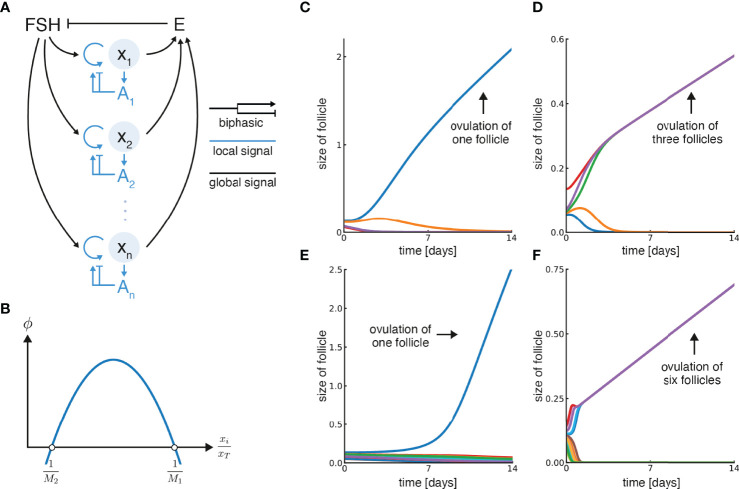
Model for ovulation number control based on a biphasic effect of local androgen. **(A)** Growth of individual follicles *x_i_* in the model is controlled by systemic feedback (circulating FSH and Estradiol, E) and local feedback (androgen concentration at each follicle, Ai). Follicles secrete estradiol and contribute to the total circulating estradiol, E, which inhibits FSH. Both local (intra-follicular) androgen and FSH control follicular growth, where FSH promotes it and androgen has a biphasic effect. **(B)** Biphasic control of follicular growth by androgen, which is proportional to the relative follicle size $\frac{x_{i}}{x_{T}}$ , is described by the function *ϕ*. Its zero-crossing points are $\frac{1}{M_{1}}$ and $\frac{1}{M_{2}}$ . **(C)** Simulation of the model with five initial follicles and *M*_1_ = 0.9, *M*_2_ = 3.5, in which a single follicle ovulates. **(D)** Simulation with five initial follicles, *M*_1_ = 2.9, *M*_2_ = 7.5, in which three follicles ovulate. **(E)** Simulation with *M*_1_ = 0.9, *M*_2_ = 10 and 15 initial follicles in which one follicle ovulates. **(F)** Simulation with *M*_1_ = 5.9, *M*_2_ = 13 and 15 initial follicles in which six follicles ovulate. Note that units of time can be rescaled by multiplying *ϕ * by a velocity constant (α), and were set so the simulation lasts 14 days by a suitable constant in each panel.

These seemingly paradoxical effects of androgen led us to posit that androgen has a biphasic (inverse-U-shaped) effect on follicle growth rate (**Figures 2A, B**).

To model ovulation and follicle growth, we developed an equation for the size of follicle *i*, denoted *x_i_*. We next describe the reasoning step by step. Since follicle cells replicate to make more of themselves, we begin with the usual $\frac{dx_{i}}{dt}∼\;x_{i}$ . We next add the fact that FSH drives the growth of follicles


$$\frac{dx_{i}}{dt}∼x_{i\;}\;FSH$$

To understand FSH as a function of time, recall that it is inhibited in the HPO axis by estrogen E (**Figure 2A**). Thus, we model

$FSH∼\frac{1}{E^{\prime }}$ , as qualitatively observed in serum hormone measurements (**Figure S2**). Estrogen is assumed to be produced by each follicle in proportion to its size. Thus, total serum estrogen goes as the sum of follicle sizes:


$$E∼\sum^{}x_{i}=x_{T}$$

Where *x_T_* is the **total size** of the follicles. We conclude that


$$\frac{dx_{i}}{dt}∼\frac{x_{i}}{x_{T}}$$

Thus, the growth rate depends on the relative follicle size, $\frac{x_{i\;}}{x_{T}}$ . If this was the only control on follicular growth, each follicle would keep growing and never be removed. We next reasoned that since each follicle makes androgen, with a high intra-follicular concentration *A_i_*, follicle growth rate also depends on the biphasic effect of androgen. Thus


$$\frac{dx_{i}}{dt}\;=\frac{x_{i}}{x_{T}}\;\phi \;\left(A_{i}\right)$$

Where *ϕ* is a biphasic function: it is negative at low and high levels of *A_i_*, and positive in between (**Figure 2B**).

To understand the dynamics of local androgen *A_i_*, we assume that each follicle produces androgen in proportion to its size,


$$A_{i}\;=\;\beta \left(t\right)x_{i}$$

where *β*(*t*) describes the time-dependent hormonal control by FSH and LH. Circulating androgen, secreted by the sum of all follicles, is observed to be nearly constant across the follicular phase except for the 2-3 days before ovulation (SI section 2, **Figure S3**). This adds a constraint to the model


$$Total\;androgen\;=A_{T}=\eta \sum_{i}A_{i}=\eta \beta \;\sum_{i}x_{i}=\eta \beta \;x_{T}\;\approx const$$

where *η* is the fraction of androgen that leaves the ovary into the circulation and is assumed to be constant. The conclusion is that $\beta \left(t\right)=\frac{A_{T}}{\eta x_{T}\left(t\right)}$ . The local androgen level is therefore, again, a function of the relative size of the follicle:


$$A_{i}\;=\frac{A_{T}}{\eta }\frac{x_{i}}{x_{T}}$$

We end up with an equation in which the growth rate of a follicle depends in a biphasic manner on its size relative to the sum of all the other follicles:


(1) $$\;\frac{dx_{i}}{dt}=\alpha \frac{x_{i}}{x_{T}}\;\phi \;\left(\frac{x_{i}}{x_{T}}\right)$$

We define the two zero crossing points of *ϕ* as $\frac{1}{M_{1}}$ and $\frac{1}{M_{2}}$ , as shown in **Figure 2B** *M*_1_ and *M*_2_ are parameters that will become important soon. For simplicity, in the simulations below we assume a parabolic form for *ϕ *, namely


(2) $$\phi \left(\frac{x_{i}}{x_{T}}\right)=\left(1-\;M_{1}\frac{x_{i}}{x_{T}}\right)\left(\;M_{2}\frac{x_{i}}{x_{T}}-1\right)$$

Other biphasic functions of the relative follicle size lead to the same qualitative conclusions. The model is similar to Lacker's model with certain alterations that change its behavior. We relate this model to Lacker’s model in Methods.

### The Model Solves ‘Choose M’ and Provides Linear Follicle Growth

This model can choose *M* follicles out of many, and provides growth at a constant velocity to the dominant follicle(s). Examples from simulations are shown in **Figure 2** and **Figure S4**. The ovulation number is determined by the biphasic function *ϕ*, and in particular by its zero-crossing points $\frac{1}{M_{1}}$ and $\frac{1}{M_{2}}$ (**Figure 2B**), as shown below.

The simulations begin with follicles with random initial sizes. A simulation where a single follicle is chosen, *M* = 1, is shown in **Figure 2C**. The dominant follicle grows and the rest shrink. In this simulation the zero-crossing parameters are *M*_1_ = 1 and *M*_2_ = 3.5.

A simulation with parameters in which *M* = 3 is chosen is shown in **Figure 2D**, namely *M*_1_ = 2.9 and *M*_2_ = 7.5. Three dominant follicles emerge and converge onto identical growth trajectories while the rest shrink. **Figures 2E, F** show simulations with a larger number of initial follicles, and different parameters, in which *M* = 1 or *M* = 6 dominant follicles arise. Note that after an initial transient, the dominant follicles approach a constant velocity in all cases.

A mathematical analysis of the model is provided in the SI (SI section 3). An intuitive way to see the main features is to solve equation (1) for the case of *M* equal-sized follicles that grow while all others die (4). Such solutions are called symmetric solutions. The relative size of the *M*growing follicles is $\frac{x_{i}}{x_{T}}=\frac{1}{M}$ . The other follicles have steady states

$\frac{x_{i}}{x_{T}}=0$ . For example, if we are interested in a symmetric solution with *M* = 3, each of the follicles is ⅓ of the total summed size. The other follicles have $\frac{x_{i}}{x_{T}}=0$ . Plugging this into Eq (1), we find that the growing symmetric follicles have a constant velocity


$$\frac{dx_{i}}{dt}=\frac{1}{M}\;\phi \left(\frac{1}{M}\right)\;=v$$

Thus


$$\begin{array}{cc} x_{i}\left(t\right)=\;v\;t\; & with\;v=\frac{1}{M}\;\phi \left(\frac{1}{M}\right) \end{array}$$

We note that a slightly more complicated model shown in the SI can provide polynomial growth to the follicle mass, as *t^q^*. This can resolve the relation between follicle diameter and mass. Cells in the follicle lie mainly on its surface in a layer that changes thickness with time, resulting in a relation between diameter and mass that lies somewhere between linear and quadratic (37) (SI section 4, **Figure S8**), so that follicle mass grows as *t^q^* where 1 < *q* < 2 when diameter grows linearly with time.

For the *M* follicles in the symmetric solution to grow in size, rather than shrink, the velocity must be positive. Thus *ϕ* must be positive. This requires that the relative size of the follicles,

$\frac{1}{M_{1}}$ , falls between the two zero points of *ϕ* so it can be in the region where the growth rate is positive. The condition for a growing solution is therefore:


$$M_{1}<M<M_{2}\;\text{condition}\;\text{for}\;\text{growing}\;\text{solution}$$

**Figure 3:**
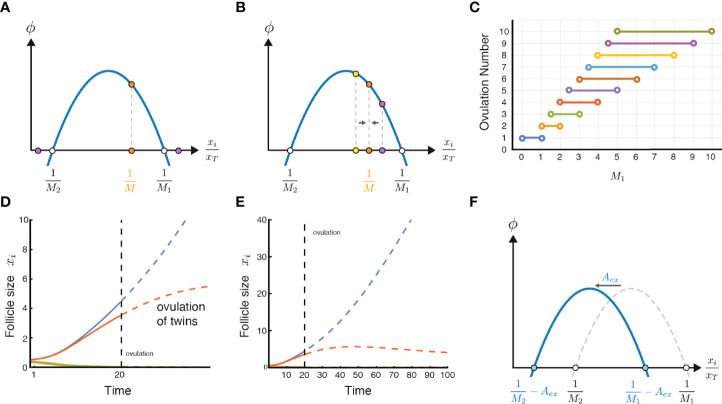
Ovulation number stability, twin ovulations, and the effect of excessive circulating androgen. **(A)** A stable solution of ovulating follicles should satisfy *M*_1_ < *M* < *M_max_*, thus $\frac{1}{M_{1}}$ is in the positive declining part of *ϕ*. **(B)** An intuitive but imprecise explanation for the criteria for the stable solution is that in the declining part of *ϕ*, deviations from the symmetric solution converge back to it, and thus the solution is stable. **(C)** Ovulation numbers as a function of *M*_1_ in the limit of *M*_2_ >> *M*_1_. When ovulation of one follicle is a possible ovulation number, it is the only stable solution. **(D)** A twin ovulation can occur even when *M* = 1 is the only stable solution, when two follicles start with a similar size and ovulate together near an unstable solution when high enough estrogen triggers the LH surge. Parameters: *M*_1_ = 0.5, *M*_2_ = 4, ovulation occurs when *x_T_* (*t*) = 4.6 *x_T_* (0) (38)(SI section 2). **(E)** Same simulation as in D continued to later times (without allowing ovulation), showing that the smaller dominant follicle eventually shrinks. **(F)** Excessive or exogenous androgen, *A_ex_*, shifts the parabola to the left, effectively changing the values of *M*_1_ and *M*_2_ and potentially disrupting ovulation.

Since *M*_1_ and *M*_2_ are parameters determined by the thresholds of local androgen for growth or death, they are assumed to be the same for all follicles, and depend on factors like androgen-receptor affinity. To obtain *M* = 3, for example, we need *M*_1_ < 3 and *M*_2_ > 3 (orange dot in **Figure 3A**). If *M>M*_2_ or *M<M*_1_, the growth rate is negative (purple dots in **Figure 3A**).

In order for the symmetric solution to be stable, there is an additional condition. If we make one of the *M* follicles slightly bigger than the rest, we want that follicle to shrink back to be equal to the rest. To see this graphically we can note that the region of stability occurs when $\frac{1}{M_{1}}$ is to the right of the maximum of *ϕ*. That is, $\frac{1}{M_{1}}$ must lie in the declining phase of the biphasic function.

To see why, imagine that we make one of the *M* follicles slightly larger than the others (**Figure 3B** purple dot). If *ϕ* is declining, the follicle grows slightly slower than the rest, and thus shrinks in relative size. The solution is stable. Likewise, if one follicle is slightly smaller (**Figure 3B** yellow dot), it grows faster, and catches up, returning to the symmetric solution.

In contrast, in the rising part of *ϕ*, to the left of its maximum, the symmetric solution is unstable. A slightly larger follicle grows faster than the rest and keeps growing, breaking the symmetric solution.

Thus, the stability criterion is that

$\frac{1}{M_{1}}>\frac{1}{M_{\max}}$ or equivalently *M* < *M_max_*. If we assume for simplicity that ϕ is a parabola, the maximum point is midway between the zeros, $\frac{1}{M_{max}}=\frac{1}{2}\left(\frac{1}{M_{1}}+\frac{1}{M_{2}}\right)=\frac{1}{2}\frac{M_{1}+M_{2}}{M_{1}M_{2}}$ , and thus

$M_{max}\;\;=2\frac{M_{1}M_{2}}{M_{1}+M_{2}}$ . Another criterion for stability for a general form of *ϕ* is $\phi \left(\frac{1}{M}\right)>\phi \left(0\right)$ ; however, since we assume *M*_1_,*M*_2_ > 0 this criterion is redundant to the positive growth criterion. The criterion for stability and positive growth together is


$$M_{1}<M<2\frac{M_{1}M_{2}}{M_{1}+M_{2}}\;condition\;for\;stable\;growing\;solution$$

If we assume for simplicity that *M*_2_ >> *M*_1_, we find


$$M_{1}<M<2M_{1}$$

The SI shows that there are other fixed-point solutions for relative follicle sizes, but the symmetric solutions are the only stable ones. It also shows that a biphasic form of *ϕ* is essential for stable solutions to the choose-M problem (SI section 3).

This model explains how setting a physiological parameter like *M*_1_ can determine the ovulation number M. This parameter, $\frac{1}{M_{1}}$ is proportional to the intra-follicle androgen concentration that is toxic to the follicle. For ovulation number *M* = 3, for example, *M*_1_ needs to be in the open interval between 1.5 and 3. For the human case *M* = 1, *M*_1_ needs to be lower than 1. **Figure 3C** shows the relation between the androgen toxicity parameter *M*_1_ and the ovulation number, when *M*_2_ is assumed to be very large, which gives the maximal range of possible ovulation numbers for a given value of *M*_1_. At *M*_1_ = 3.4, for example one can have 4, 5 or 6 ovulating follicles in the model.

### Twin Ovulations in the Model

The present model provides insight into the occurrence of twin ovulations. Such dizygotic (non-identical) twins occur in unassisted human ovulations with a frequency of about 1 out of 90 pregnancies (39, 40). Monozygotic twins, which originate after ovulation, are beyond the scope of this model.

In the model, dizygotic ovulations can occur even if the only stable solution is *M* = 1. If two follicles happen to start the race with very similar initial sizes, they grow together (**Figure 3D**). Given enough time, the smaller follicle would eventually die in the model because *M* = 2 is unstable (**Figure 3E**). But given the limited duration of the follicular phase due to the estradiol levels that trigger the LH surge, it sometimes happens that the second follicle is so close in size to the dominant follicle that it also makes it to the LH surge. In this case both follicles ovulate (**Figure 3D**).

The probability for triplets and quadruplets in this picture drops exponentially because it is increasingly unlikely to have three or four dominant follicles with such nearly identical initial sizes that can keep together throughout the process. This is similar to the observed drop in frequencies: Naturally, dizygotic twins occur in about one in 90 pregnancies, triplets in about one in 8000 pregnancies, and quadruplets in about one in 400,000 pregnancies (39, 41).

### The Model Can Explain Qualitative Follicle Dynamics in PCOS

The present mechanism also explains how excessive chronic circulating androgen might interfere with ovulation, as occurs in many cases of PCOS. High levels of external androgen effectively shift the biphasic curve to the left (**Figure 3F**). High enough levels prevent growing solutions and no ovulations can occur.

The model also relates to the dynamics of follicles in PCOS. These dynamics have an interesting history with respect to modelling. In the 1980’s it was believed that in PCOS follicles become arrested and persist at an intermediate size. Lacker’s model (4) and later variants (28) showed such a steady-state of growth arrest for certain parameters. Mathematically, this growth arrest solution can be seen from the symmetric solution of Lacker’s model (Methods).

**Figure 4:**
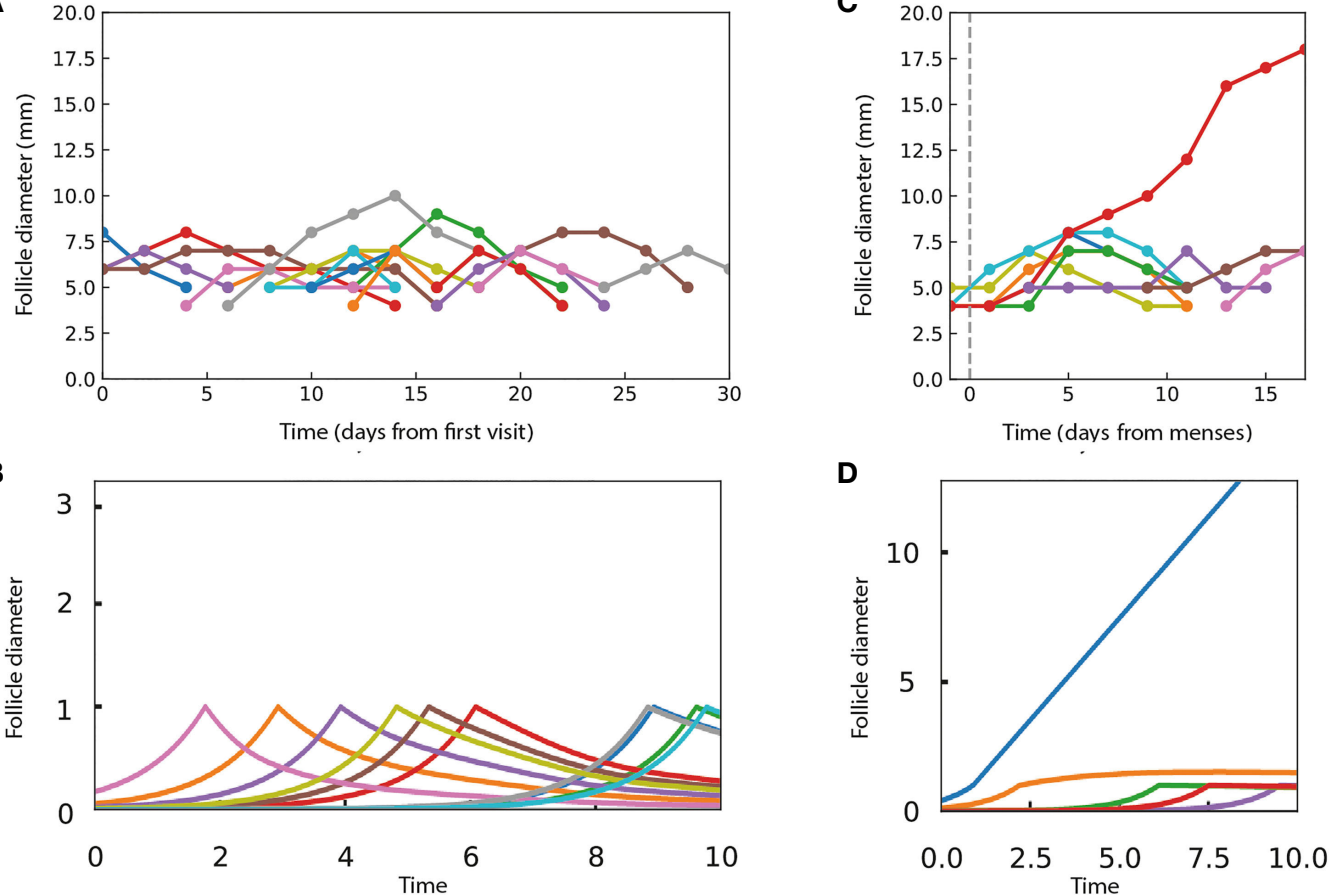
The present model can provide dynamics that are qualitatively similar to ultrasound experiments in women with and without PCOS. **(A)** Data from Jarrett et al. on follicle diameter in a participant with PCOS shows continual entry of follicles, which stop growth at a typical size and then shrink. **(B)** Dynamics in simulations in which follicles grow independently until they reach a critical size of *x_c_* = 1 and then compete according to the androgen model. Parameters do not allow ovulation, with M1 = 0.9, M2 = 10, external androgen *A_ex_* = 5 and α~0.005. **(C)** Data from Jarrett et al. on follicle dynamics in a control participant without PCOS, showing a single dominant follicle that ovulates and gradual shrinkage of the other follicles. The dashed line indicates the start of the follicular phase. **(D)** A single dominant follicle is seen in simulations as in B, but with parameters that allow ovulation, M1 = 0.9, M2 = 10, *A_ex_* = 0, and α = 1. In the simulations, follicle size is in arbitrary units.

The assumption of persistent growth-arrest in PCOS recently changed when longitudinal ultrasound in humans was reported by Jarrett et al. (17). PCOS involves more follicles than normal ovulation. These follicles grow, reach a typical size of about 7.5mm, and then decline (**Figure 4A**). There are no persistent growth-arrested follicles in PCOS according to the available data.

Notably, the present model differs from Lacker’s model in having no growth arrested steady-states. Instead, the symmetric solutions show either growth or decline with constant velocity, due to the scaling property of the model. Mathematically, $\frac{dx_{i}}{dt}=\frac{x_{i}}{x_{T}}\phi \left(\frac{x_{i}}{x_{T}}\right)$ provides a constant velocity for a symmetric solution $\frac{dx_{i}}{dt}=\frac{1_{}}{M_{}}\phi \left(\frac{1_{}}{M_{}}\right)$ , either positive (ovulation) or negative (decline).

We asked to what extent does the present model provide dynamics similar to those observed experimentally in humans. Due to its simplicity, it is unlikely that the model can give quantitatively precise agreement with experiments, and instead we aimed at a qualitative agreement. To compare the model to simulations, we adjusted the model to allow follicles to enter the cycle at different times as experimentally observed (**Figure 4A**). To do so, we assume that follicles begin to grow independently and exponentially at random times from a small initial size


(3) $$\begin{array}{cc} \frac{dxi}{dt}=\gamma x_{i} & while\;x_{i}<x_{c} \end{array}$$

When a follicle reaches a critical size, *x_c_* it undergoes a developmental switch. Thereafter, the follicle obeys the present model’s competition dynamics, even if it shrinks below *x_c_*,


(4) $$\frac{dxi}{dt}=\alpha \frac{x_{i}}{x_{T}}\phi \left(\frac{x_{i}}{x_{T}}+A_{ex}\right)$$

where *A_ex_* is external androgen and *x_T_* = *Σx_i_* is the sum over all follicles regardless of size. In simulations, we used a parabolic form for *ϕ* given by Eq 2.

We simulated PCOS by adding high androgen levels that effectively shift *M*_1_and *M*_2_ to low numbers that prohibit ovulation. The simulations (**Figure 4B**) show follicles that grow to size *x_c_* and then shrink, similar to the experimental ultrasound data for women with PCOS (**Figure 4A**).

To simulate ovulatory conditions, we use M_1_=0.9, M_2_ = 10 with no external androgen. We also set a smaller number of follicles to enter the cycle per unit time, as observed (**Figure 4C**). The model displays a dominant follicle, typically one that starts early in the follicular phase of the cycle (**Figure 4D**). The dominant follicle grows with a constant velocity. The subdominant follicles grow, reach the critical size, and then gradually shrink, qualitatively similar to the experimental data (**Figures 4C, D**).

We were not able to obtain a closer fit to the experimental data with other model parameters. This indicates that a more complex model is required if precise fits to experiments are the goal.

The experimental data of Jarrett et al. also shows follicles that grow and then shrink during the luteal phase of the cycle, in both PCOS and non-PCOS participants. The present model concerns the follicular phase. The luteal phase involves a different hormonal profile, including progesterone produced by the corpus luteum and altered levels of FSH, LH and estrogen, which may require additional modelling.

We conclude that the proposed androgen-based mechanism leads to a mathematical model that can explain some of the qualitative features of follicle dynamics in normal and PCOS-like situations.

## Discussion

We presented a physiological mechanism and mathematical model for the control of ovulation number in mammals. In this mechanism a specific number of follicles are chosen to ovulate whereas others die by means of a circuit in which follicles measure their relative sizes. The circuit has two signals: systemic control by FSH together with a new follicle-centric androgen signal. This local androgen signal inhibits a follicle’s growth if the relative size of the follicle is too large or too small. The model provides species-specific ovulation numbers regulated by the local androgen toxicity threshold. The model explains the observed constant velocity of the dominant follicle size, and explains the disruption of ovulation and decline of non-ovulating follicles in PCOS caused by high circulating androgen levels.

The mechanism is based on a biphasic (inverse U-shaped) effect of local androgen. Both high and low levels inhibit follicular growth. A biphasic function of follicle size was assumed in Lacker’s classic model of ovulation without a physiological basis; here we suggest that androgen provides a physiological basis.

Biphasic or non-monotonic behavior occurs in other physiological settings including endocrine, immune and neuronal systems (42, 43). Biphasic control of growth was suggested by Karin et al. (42, 43) to act as a mutant-resistance mechanism for mutants that mis-sense a feedback signal. In the present model, the biphasic effect plays a dynamical role, eliminating follicles that are too large or too small relative to the others.

The present model has a scaling property in which follicle growth rate depends only on its relative size compared to the sum of all other follicle sizes. Follicle sizes converge to symmetric solutions where all dominant follicles have equal sizes. This symmetry and scaling provides a constant growth velocity to the dominant follicles. Such a constant velocity is observed in longitudinal ultrasound studies of ovulation (1, 17). This differs from Lacker’s model and its variants, which do not scale each follicle to the total follicular mass, and as a result predict that the dominant follicles grow super-exponentially and reach infinite size at a finite time.

The present model has another key difference from Lacker’s model and its variants (28) in terms of the follicular growth profiles in PCOS. The earlier studies assumed that follicles in PCOS arrest and persist at a fixed size, and their models provide such a fixed-point solution. However, recent ultrasound measurements (17) indicate that in PCOS follicles stop growing prematurely and then shrink, rather than persisting at an intermediate size. The present model does not allow a persistent growth-arrest solution. Instead, it can provide turnover of follicles that grow and then shrink, similar to the experimental trajectories. This turnover is seen by simulating a process in which small follicles grow independently at first and then start competing according to the androgen mechanism at a certain developmental time or critical size.

Growth and shrinkage of follicles was also addressed in a recent mathematical model by Lange et al. (44) of anovulatory waves in cows. In these waves, follicles grow and then shrink without ovulating. To fit this behavior, Lange et al. introduced a term in which the follicular death rate increased linearly with time; this term lacks a known physiological basis, as stated by the authors, who intended to provide a minimal model to fit data rather than a physiological mechanism. The present androgen mechanism may be able to describe such anovulatory processes of follicular growth and shrinkage.

The proposed mechanism can be extended in several ways. One can consider competition between neighboring follicles, such as paracrine interactions and competition over blood vessels. It can also be extended by considering follicular developmental stages. This can be done by using the models of Clement, Monniaux and colleagues (5, 6, 14, 19, 45–50), which provide a continuous and deterministic description of follicle development based on the hormonally regulated partition of granulosa cells into different cell states: proliferation, differentiation and apoptosis. This detailed framework can help to explore the present mechanism by making the transition probabilities between cell states depend on intra-follicular androgen levels in a biphasic manner. Other extensions include changes that occur with age (51) and maturation and ageing of follicles (27, 52), as well as hormone-induced changes in pituitary gonadotroph cell mass, in analogy to recent models of the HPA axis (53–55).

The present model assumes that all follicles have the same response parameters to hormones and the same intrinsic growth rates. This can be extended to the case of heterogeneous follicle parameters using the methods of Chávez-Ross et al., which analyzed heterogeneity in Lacker’s model (28). Noise can also be added to the model. These extensions can break the strict ordering of the follicle sizes throughout their growth.

The present model suggests several experimental tests that can refute it. It would be important to explore in detail whether intra-follicular androgen has a biphasic effect on follicle growth rate. Such experiments can in principle be done *in vitro*. One can provide androgen at different concentrations and measure the effects on follicular-cell growth and death rates. These experiments can thus map the function *ϕ*.

*In vivo*, one can measure intra-follicular androgen levels from different follicles and relate these levels to single-cell gene expression from the same follicles. The model predicts that androgen concentration in a given follicle will have a biphasic effect on growth, differentiation and artesia expression programs in the theca and granulosa cells.

Another possible experiment is measurement of the intra-follicular androgen levels alongside the follicle diameter and its granulosa cell number and mass. Our theory predicts that intra-follicular androgen will be proportional to the relative size of each follicle, rather than to the absolute size.

One can use these approaches to compare *ϕ* from species with different ovulation numbers. The model predicts that the androgen thresholds for growth and toxicity, which are described by the zero points of *ϕ* denoted *M*_1_ and *M*_2_, will vary between species to provide the species-specific range of ovulation numbers. The model can also be tested by performing detailed longitudinal measurements of follicle sizes in species with different ovulation numbers using ultrasound (42, 55). The simplicity of the model suggests a qualitative comparison to data, and probably precludes precise quantitative fits. Future improvements can aim to provide such quantitative fits.

This study provides a perspective on PCOS in which the disorder is a fragility (exposed by modern conditions) of an essential androgen-based mechanism for ovulation number control. The biphasic androgen mechanism ensures that the ovulation number is in a specific range, which can be important for fitness of each species. The price of such a control mechanism is that excessive androgen can disrupt ovulation. Conditions of chronic excess ovarian androgen were probably rare historically before the rise of insulin resistance and obesity. We hope that the present framework will help to improve our understanding of ovulation, PCOS and infertility.

## Methods

### Simulations of the Follicular Growth Model

Initial conditions were *N* follicles with sizes given by random numbers from a uniform distribution between 0.05 and 0.15. Ovulation was simulated by the time when total follicle size *x_T_* exceeded a threshold. Based on experiments (38), we set the threshold at 4.6 times the initial *x_T_* (SI section 2). We used the solver scipy.integrate.ode (scipy version 1.7.0) of python 3.8.11. Code is available at github.com/michalshilo6/OvulationNumberControl.

To simulate continual entry of follicles in **Figure 4**, we used Eq 3-4. We generated a set of random times *t_i_* throughout the follicular phase, and started follicles at *t* = 0 with initial sizes *x*_*i*_=*x*_*c*_ *e* ^−*γt*_*i*_^ so that they reach *x_c_* at times *t_i_*. Parameters were ′*γ* = 1 and *x_c_* = 1; times and sizes can be scaled by appropriate parameter changes. External androgen *A_ex_* shifts the biphasic function to the left. The larger M2, the less sensitive the dominant follicle dynamics to the entry of new follicles.

### Relation to Lacker’s Model

Lacker’s model in its most commonly used form (4) is, upto parameters which can be rescaled away, *dx_i_*/*dt* = *x_i_ g*(*x_i_*,*x_t_*) with *g*(*x_i_*,*x_t_*) = 1 – (*x_T_* – *M*_1_ *x_i_*) (*x_T_* – *M*_2_ *x_i_*) and *x_T_* = *Σ x_i_*. Symmetric solutions are obtained when M follicles have equal sizes *x__i_* = *x__T_*/*M* and the rest have *x_i_* =0. The symmetric solution obeys $\frac{dx_{T}}{dt}=x_{T}+\mu x_{T}^{3}$ with $\mu =-\left(1-\frac{M_{1}}{M}\right)\left(1-\frac{M_{2}}{M}\right)$ . In ovulatory conditions, /mu is positive, and hence the dominant follicles diverge to infinite size at finite time. In anovulatory conditions /mu is negative and follicles reach growth arrest at a steady-state size $x_{i}=\frac{1}{\sqrt{\left|\mu \right|}}$.

The present model (Eq 1-2) can be obtained as follows. First delete the 1 from the function g, resulting in $\frac{dx_{i}}{dt}=x_{i}x_{T}^{2}\left(1-M_{1}\frac{x_{i}}{x_{T}}\right)\left(M_{2}\frac{x_{i}}{x_{T}}-1\right)$ . This is the present model times $x_{T}^{3}$ . Time can be transformed to a new variable τ such that $d\tau /dt=x_{T}^{3}$ , so that $\frac{dx_{i}}{d\tau }=u_{i}\phi \left(u_{i}\right)$ with $u_{i}=\frac{x_{i}}{x_{T}}$ and *ϕ*(*u_i_*) = (1 – *M*_1_ *u_i_*) (*M*_2_ *u_i_* - 1). This is the present model with a transformed timescale. The transformed timescale changes the asymptotic growing solutions from singular with finite-time divergence in Lacker’s model to a constant velocity in the present model.

The constant factor 1 in Lacker’s function g provides exponential growth to small follicles. It is also responsible for the growth-arrested steady-states in Lacker’s model by balancing the parabolic part of g. In the present model of **Figure 4**, exponential growth occurs for small follicles but stops when follicles cross a developmental threshold at a critical size. Thereafter, there is no term to balance the biphasic function *ϕ* and hence no growth-arrested solutions.

## Data Availability Statement

The original contributions presented in the study are publicly available. This data can be found here: https://github.com/michalshilo6/OvulationNumberControl.

## Author Contributions

Conceptualization: MS and UA; Data curation: MS and UA; Formal analysis: all authors; Investigation: all authors; Methodology: MS and UA; Software: MS and AM; Supervision: UA; Visualization: MS and AM; Writing- original draft: MS and UA; Writing- review & editing: all authors. All authors contributed to the article and approved the submitted version.

## Funding

This work was supported by funding from Cancer Research UK (C19767/A27145), and by internal funding of the Weizmann institute of science.

## Conflict of Interest

The authors declare that the research was conducted in the absence of any commercial or financial relationships that could be construed as a potential conflict of interest.

## Publisher’s Note

All claims expressed in this article are solely those of the authors and do not necessarily represent those of their affiliated organizations, or those of the publisher, the editors and the reviewers. Any product that may be evaluated in this article, or claim that may be made by its manufacturer, is not guaranteed or endorsed by the publisher.
