# Supplementary material for: A Mechanism for Ovulation Number Control
Source: Front Endocrinol (Lausanne). 2022 Jul 14;13:816967. doi: 10.3389/fendo.2022.816967 (PMC9329923; doi:10.3389/fendo.2022.816967)
Supplement: Supplementary file 1 [file DataSheet_1.pdf]

A mechanism for ovulation number control: Supplementary information

Table of Contents

S1. Diameter of dominant follicle during the follicular phase 1

S2. Serum androgen levels over the follicular phase 2

S3. Analytical solution of model fixed points and stability 4

S4. Polynomial dependence of follicle mass on time.. 15

S1. Diameter of dominant follicle during the follicular phase

Ultrasound measurements in women indicate that the diameter of the dominant follicle rises approximately linearly with time during the follicular phase, as can be seen in Fig 1D and in Fig S1. Similar results are found in ref [1].

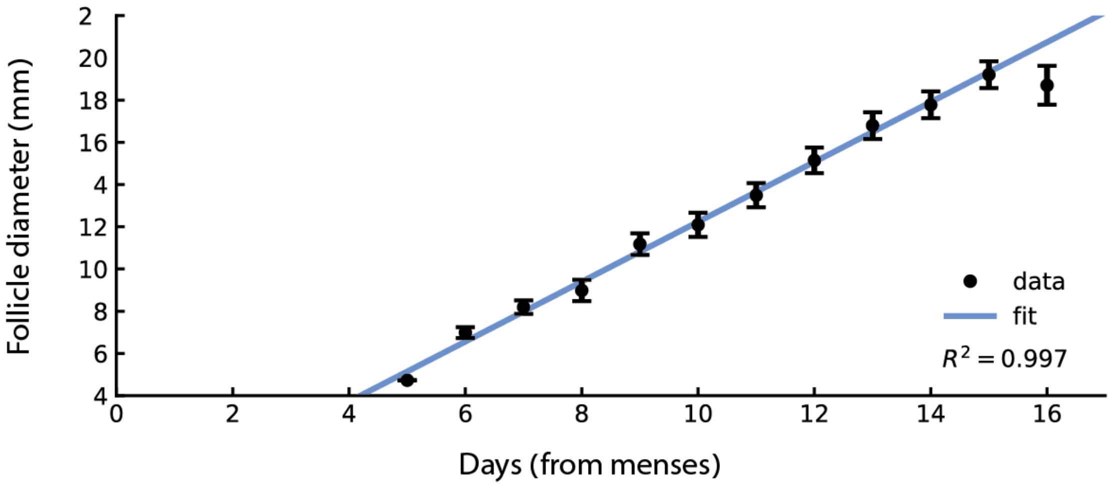

Figure S1. Follicles grow linearly with time Diameter of the dominant follicle in the ovulatory wave of women with three waves of follicular development grows with an approximately constant velocity, measured by ultrasound and adapted from [2]. Regression line is shown. Figure 1D presents the same data for the ovulatory wave of women with two waves of follicular development.

S2. Serum FSH, estradiol and androgen levels over the follicular phase

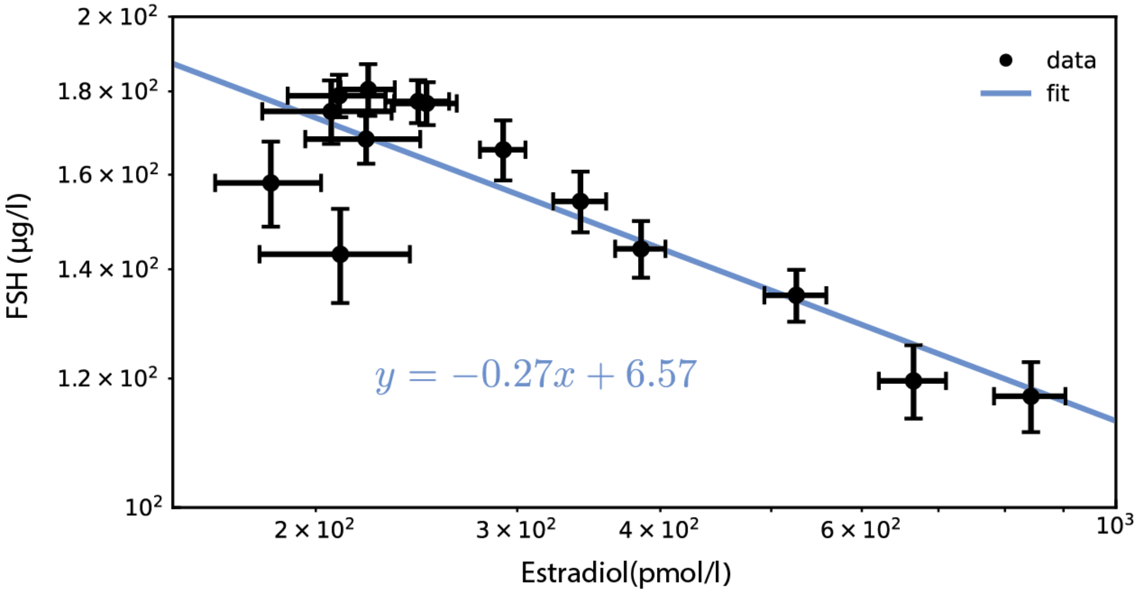

Figure S2. FSH drops with estradiol Serum FSH levels scale with estradiol with a log-log slope of -0.27 ± 0.04 during most of the follicular phase. Measurements shown are from day -15 to day -2 relative to the LH surge, after which FSH starts to rise fast as a result of change of sign of estradiol control of gonadotropins. Data is adapted from [3].

Androstenedione and Testosterone are both relatively constant over the follicular phase, and rise during the LH surge (2-3 days before ovulation). An experiment that inhibits adrenal androgen production using dexamethasone enables one to estimate the ovarian contribution to serum androgens[4]. During the first 12 days of the follicular phase, Testosterone changes by a factor of 1.55 ± 0.15 and Androstenedione changes by a factor of 1.90 ± 0.22. This is a small change relative to the estimated change in estradiol levels which is 4.6 ± 0.6 fold, and should represent the change in x_T.

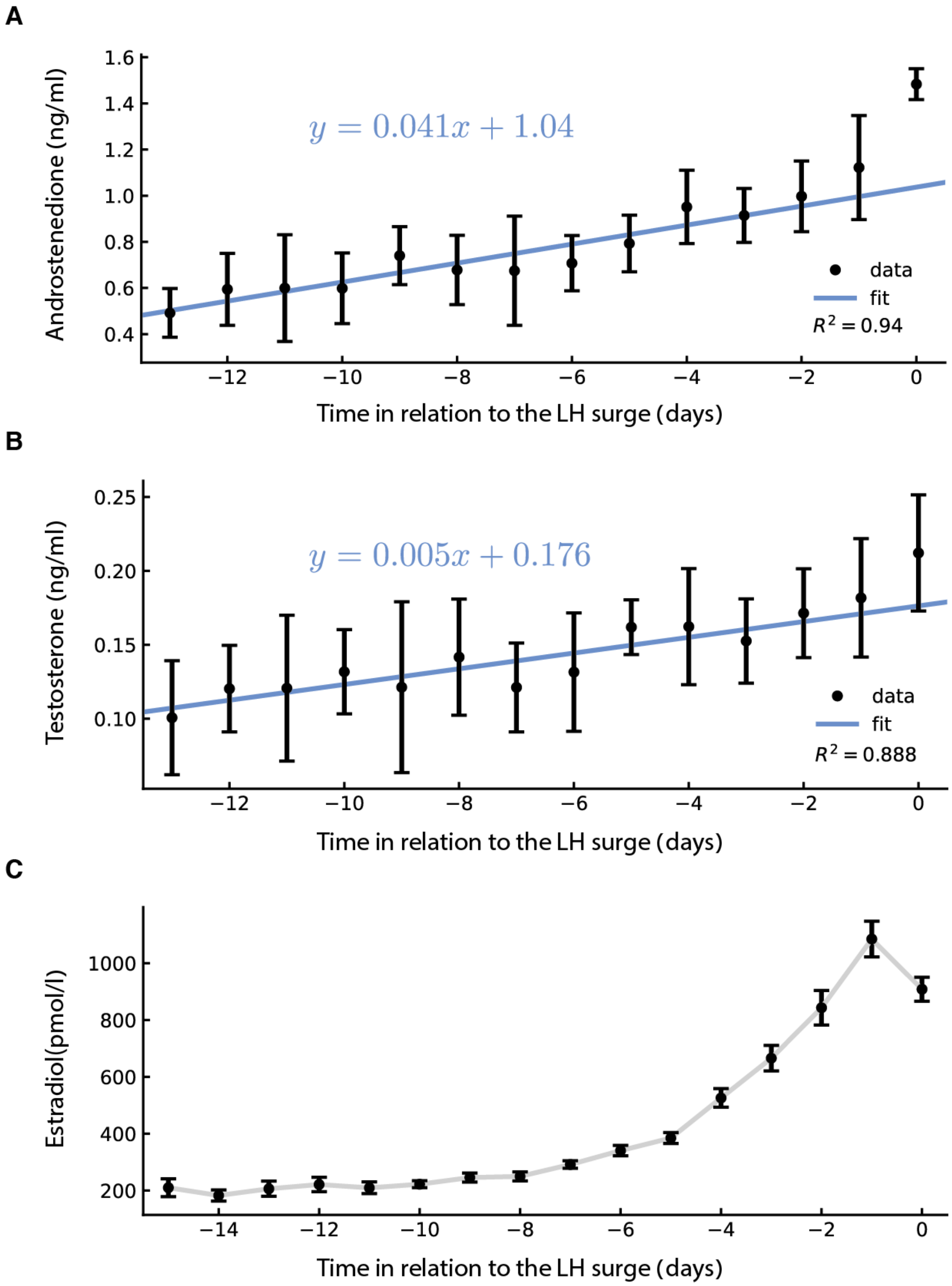

Figure S3. Estradiol changes more than androgen during the follicular phase (A) Androstenedione secreted from the ovaries grows linearly in time, with a slope of *0.041* $\pm$ *0.005* and intercept of *1.04* $\pm$ *0.04*, measured in serum of women after suppression of the adrenal cortex. Regression line was calculated from the beginning of the cycle to day -2 relative to the LH

surge. Adapted from [4], where the cycle length was normalized so that the first day of the cycle is day -13 with relation to the LH surge. (B) Testosterone secreted from the ovaries grows linearly in time, with a slope of *0.005* $\pm$ *0.001* and intercept of *0.176* $\pm$ *0.007*, measured in serum of women after suppression of the adrenal cortex. Regression line was calculated from the beginning of the cycle to day -2 relative to the LH surge. Adapted from [4], where the cycle length was normalized so that the first day of the cycle is day -13 with relation to the LH surge. (C) Estradiol changes more dramatically through the follicular phase. Estradiol shows a growth of *4.6* $\pm$ *0.6* fold from a minimal value of 180 pmol/l in the second day of the cycle it grows to*840* pmol/l at day -2 relative to the LH surge, after which FSH starts rising rapidly due to positive control of estradiol, which our model doesn't describe. Adapted from [3], where the cycle length was normalized so that the first day of the cycle is day -15 in relation to the LH surge.

S3. Analytical solution of model fixed points and stability

S3.1. Description of follicular growth rate using differential equations

Let us assume that N follicles start growing at the onset of the follicular phase. Following Lacker, we assume the size of a follicle i reflects its contribution to the circulatory estradiol:

$$x_i = a_E E_i$$

We assume Estradiol levels are controlled by the mass of granulosa cells so that

$$E_T = \sum E_i = \sum a_E x_i = a_E x_T$$

FSH is produced by the pituitary P and is inhibited by estradiol E_T[5]:

$$\frac{dFSH}{dt} = a_{FSH}\frac{P}{E_T} - b_{FSH}FSH$$

In quasi-steady-state $FSH = \frac{a_{FSH}}{b_{FSH}}\frac{P_{st}}{E_T} = \frac{a_{FSH}}{b_{FSH}a_E}\frac{P_{st}}{x_T}$

FSH promotes follicular growth, and assuming androgen controls follicular growth through some function $\phi(A_i)$, we can write the following equation:

$$\frac{dx_i}{dt} = a_x x_i\, FSH\, \phi(A_i)$$

We assume larger follicles produce more androgen:

$$A_i = g(x_T)\, x_i$$

With some function g of the total follicular size (which correlates with Estradiol and FSH). The levels of circulating androstenedione are relatively constant (comparing to follicular mass) through the follicular phase, increasing by about 1.5-2-fold (Fig S3) whereas the total follicular mass increases by a larger factor, about 4-5-fold, hence:

$$A_T = \sum A_i = g(x_T) \sum x_i = g(x_T)\, x_T \approx constant$$

$$g(x_T, t) = \frac{A_T}{x_T}$$

And we can write:

$$\frac{dx_i}{dt} = \alpha \frac{x_i}{x_T}\, \phi\left(A_T \frac{x_i}{x_T}\right)$$

$$\alpha = a_x \frac{a_{FSH}}{b_{FSH}} P_{st}$$

In the main text the factor α is included in ϕ. We obtain a model for follicular growth which depends only on the *relative size of the follicle*. This means that the follicle growth rate is invariant with respect to multiplying all follicle sizes by a constant.

In the main text figures we used a parabolic dimensionless form for:

$$\phi(A_i) = (1 - M_1 \gamma \frac{A_i}{A_T})(1 - M_2 \gamma \frac{A_i}{A_T}) = (1 - M_1 \frac{x_i}{x_T})(1 - M_2 \frac{x_i}{x_T})$$

The physiological interpretation of M_1 is that the threshold of local androgen toxicity is $\frac{A_T}{\gamma M_1}$. The physiological interpretation of M_2 is that the threshold for growth of the follicle in terms of local androgen concentration is $\frac{A_T}{\gamma M_2}$. Since growth occurs at lower concentrations than toxicity, we have $M_2 > M_1$. We will continue to use this form of ϕ in the next sections.

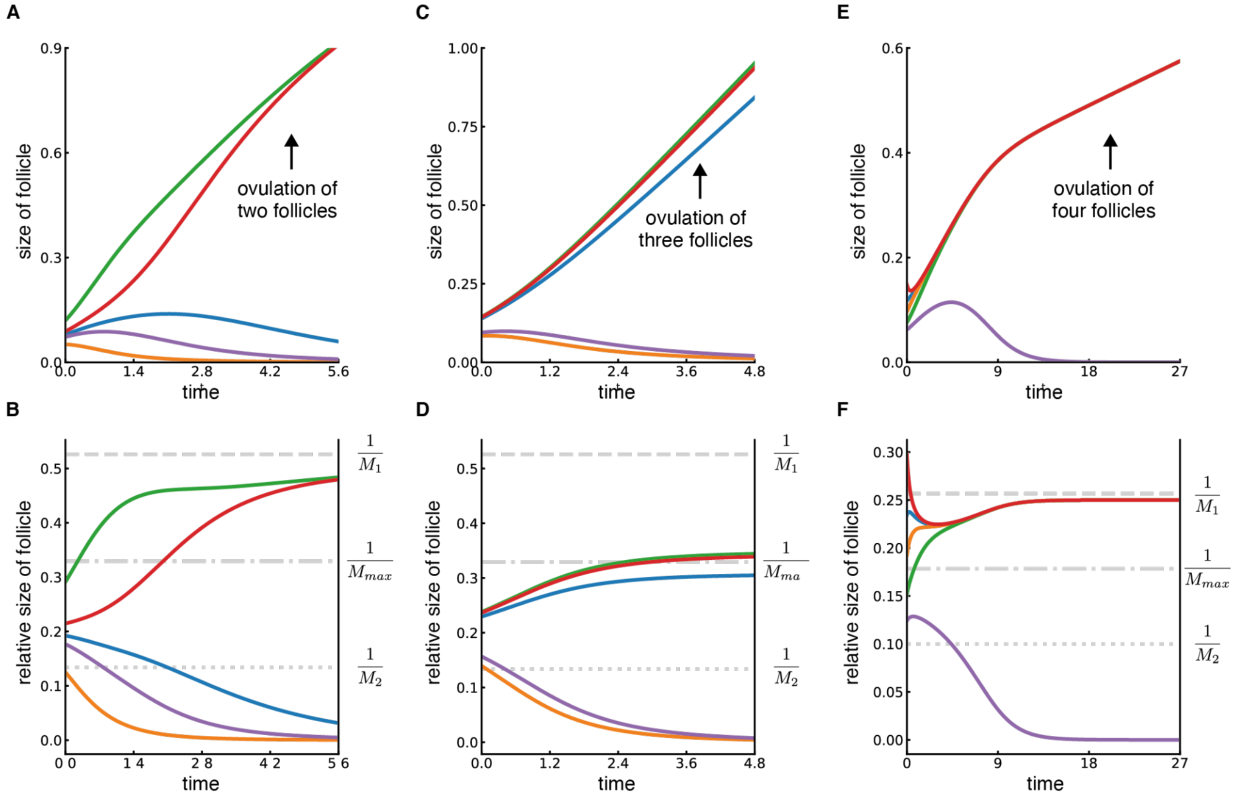

Figure S4. Simulation of the model for ovulation number control.

(A) Simulation follicular growth with five initial follicles and $M_1 = 1.9$, $M_2 = 7.5$, in which two follicles ovulate. The sizes of the follicles (x_i) are plotted. (B) Relative sizes of the follicles ($\frac{x_i}{x_T}$) from the simulation in A. (C) Simulation follicular growth with five initial follicles and $M_1 = 1.9$, $M_2 = 7.5$, in which three follicles ovulate. The sizes of the follicles (x_i) are plotted, with relative sizes in (D). (E) Simulation follicular growth with five initial follicles and $M_1 = 3.9$, $M_2 = 10$, in which four follicles ovulate. The sizes of the follicles (x_i) are plotted, with relative sizes in (F).

S3.2. Stability analysis reveals stable ovulations are symmetrical solutions in which ovulatory follicles grow linearly in time

In order to understand the behavior of follicles in our model we would like to examine the fixed points of the system. However, since we are modeling ovulation, where follicles are growing, there are no fixed-points in the follicular size space. To make progress, we can use the property of the model that follicular growth depends only on the relative follicular size, to transform our equations to the relative follicular size space, and find stable fixed points of growing follicles. The derivation that follows is similar to that of Akin and Lacker for the Lacker model[6].

We denote the relative size of follicle i by $u_i = \frac{x_i}{x_T}$. We can write:

$$\frac{dx_i}{dt} = u_i \phi(u_i)$$

Next, we convert the set of equations for the N follicles from absolute size space to relative size space:

$$\frac{du_i}{dt} = \frac{\partial u_i}{\partial x_i} \cdot \frac{dx_i}{dt} + \frac{\partial u_i}{\partial x_T} \cdot \frac{dx_T}{dt} = \frac{1}{x_T} u_i \phi(u_i) - \frac{x_i}{x_T^2} \sum_{j=1}^{N} u_j \phi(u_j)$$

$$\frac{du_i}{dt} = \frac{1}{x_T} \left(u_i \phi(u_i) - u_i \sum_{j=1}^{N} u_j \phi(u_j) \right)$$

To eliminate the $\frac{1}{x_T}$ term, we define a new time variable τ:

$$\tau = \int_0^t \frac{1}{x_T} dt \rightarrow \frac{dt}{d\tau} = x_T$$

$$\frac{du_i}{d\tau} = \frac{du_i}{dt} \cdot \frac{dt}{d\tau} = u_i \phi(u_i) - u_i \sum_{j=1}^{N} u_j \phi(u_j)$$

which is an equation with only the u_i variables.

Now we can find the fixed points of our system in the relative size space, by setting the derivative $\frac{du_i}{d\tau}$ to zero:

$$u_i \phi(u_i) - u_i \sum_{j=1}^{N} u_j \phi(u_j) = 0$$

Then either u_i = 0, or:

$$\phi(u_i) = \sum_{j=1}^{N} u_j \phi(u_j)$$

Thus, at the fixed point, $\phi(u_i) = c = \sum_{j=1}^{N} u_j \phi(u_j)$ is the same constant for each i where $u_i \neq 0$.

One family of solutions can be obtained when all the non-zero follicle have the same absolute size, thus they share also their relative size and their value for ϕ. We will denote this as the **symmetric solution**. Another family of solutions can be obtained when non-zero follicles with different sizes share the same value of ϕ. We call this the **semi-symmetric solution**.

Let's examine the symmetric solution first, because it's simpler and we can obtain the criteria for stability of the fixed points without knowing the form of ϕ. Assuming we have M equal-sized follicles with non-zero sizes $(x_1, \dots, x_M)$, and $N - M$ follicles who have gone through atresia and have size 0, $(x_{M+1}, \dots, x_N)$, the relative size of the non-zero follicles is:

$$u_i = \frac{1}{M}$$

In order to find when a symmetric solution is stable, we perturbate the system from the fixed point by $\delta = (\delta u_1, \delta u_2, \dots, \delta u_N)$. We want to know whether the perturbation grows or shrinks.

$$\frac{d(u_i+\delta)}{d\tau}_{\left(u_i=\frac{1}{M}\right)} = \frac{du_i}{d\tau}_{\left(u_i=\frac{1}{M}\right)} + \frac{d\delta}{d\tau}_{\left(u_i=\frac{1}{M}\right)} = \frac{d\delta}{d\tau}_{\left(u_i=\frac{1}{M}\right)}$$

$$\frac{d\delta}{d\tau}_{\left(u_i=\frac{1}{M}\right)} = \frac{d(u_i+\delta)}{d\tau}_{\left(u_i=\frac{1}{M}\right)} = \frac{du_i}{d\tau}_{\left(u_i=\frac{1}{M}\right)} + \frac{\partial\left(\frac{du_i}{d\tau}\right)}{\partial u_1}\delta u_1 + \frac{\partial\left(\frac{du_i}{d\tau}\right)}{\partial u_2}\delta u_2 + \cdots + \frac{\partial\left(\frac{du_i}{d\tau}\right)}{\partial u_N}\delta u_N$$

$$\frac{\partial\left(\frac{du_i}{d\tau}\right)}{\partial u_i} = (1-u_i)\phi(u_i) + (u_i - u_i^2)\phi'(u_i) - \sum_{j=1}^{N} u_j\phi(u_j)$$

$$\frac{\partial\left(\frac{du_i}{d\tau}\right)}{\partial u_i}_{\left(u_i=\frac{1}{M}, symmetric\ solution\right)} = \left(\frac{1}{M} - \frac{1}{M^2}\right)\phi'\left(\frac{1}{M}\right) - \frac{1}{M}\phi\left(\frac{1}{M}\right) \equiv a$$

$$\frac{\partial\left(\frac{du_i}{d\tau}\right)}{\partial u_i}_{(u_i=0, symmetric\ solution)} = \phi(0) - \phi\left(\frac{1}{M}\right) \equiv b$$

$$\frac{\partial\left(\frac{du_i}{d\tau}\right)}{\partial u_j}_{j\neq i} = -u_i\left(\phi(u_j) + u_j\phi'(u_j)\right)$$

$$\frac{\partial\left(\frac{du_i}{d\tau}\right)}{\partial u_j}_{(u_i=0, j\neq i, symmetric\ solution)} = 0$$

$$\frac{\partial\left(\frac{du_i}{d\tau}\right)}{\partial u_j}_{\left(u_i=u_j=\frac{1}{M}, j\neq i, symmetric\ solution\right)} = -\frac{1}{M}\phi\left(\frac{1}{M}\right) - \frac{1}{M^2}\phi'\left(\frac{1}{M}\right) \equiv c = a - \frac{1}{M}\phi'\left(\frac{1}{M}\right)$$

$$\frac{\partial\left(\frac{du_i}{d\tau}\right)}{\partial u_j}_{\left(u_i=\frac{1}{M}, u_j=0, j\neq i, symmetric\ solution\right)} = -\frac{1}{M}\phi(0) \equiv d$$

Thus, the Jacobian is:

$$J = \left(\begin{array}{ccccc|ccc} a & & & & c & & & \\ & a & & & & & d & \\ & & \ddots & & & & & \\ & & & a & & & & \\ c & & & & a & & & \\ \hline & & & & & b & & 0 \\ & & 0 & & & & \ddots & \\ & & & & & 0 & & b \end{array}\right)$$

This is a block matrix, so the eigen values can be calculated for the blocks J_1, J_2:

$$J_1 = \begin{pmatrix} a & & & & c \\ & a & & & \\ & & \ddots & & \\ & & & a & \\ c & & & & a \end{pmatrix} \quad , \quad J_2 = \begin{pmatrix} b & & 0 \\ & \ddots & \\ 0 & & b \end{pmatrix}$$

The eigenvectors of J_1 are:

$v_1 = \begin{pmatrix} 1 \\ \vdots \\ 1 \end{pmatrix} = I_1$ with eigenvalue of

$$\lambda_1 = a + (M-1)c = -\phi\left(\frac{1}{M}\right)$$

With multiplicity of 1.

$v_{2,\dots,M} = \begin{pmatrix} 1 \\ -1 \\ 0 \\ \vdots \\ 0 \end{pmatrix}, \begin{pmatrix} 1 \\ 0 \\ -1 \\ \vdots \\ 0 \end{pmatrix}, \dots, \begin{pmatrix} 1 \\ 0 \\ 0 \\ \vdots \\ -1 \end{pmatrix}$ with eigenvalue of

$$\lambda_2 = a - c = \frac{1}{M}\phi'\left(\frac{1}{M}\right)$$

with multiplicity of $M - 1$.

The eigenvector of J_2 is $\lambda_3 = b = \phi(0) - \phi\left(\frac{1}{M}\right)$ with multiplicity of $N - M$.

In order for the symmetrical solution to be a stable solution the eigenvalues of the Jacobian matrix should be negative, hence:

$$\lambda_1 = -\phi\left(\frac{1}{M}\right) < 0 \rightarrow \phi\left(\frac{1}{M}\right) > 0$$

Notice the eigenvector of this eigenvalue is irrelevant to the framing of the problem where $\sum_i u_i = 1$, as it represents a change in $\sum_i u_i = 1$. However, in order for the stable solutions to represent solutions of symmetrically growing follicles, $\phi\left(\frac{1}{M}\right)$must be positive.

$$\lambda_2 < 0 \rightarrow \phi'\left(\frac{1}{M}\right) < 0$$

Finally, $\lambda_3 < 0 \rightarrow \phi(0) < \phi\left(\frac{1}{M}\right)$.

To summarize, the stability criteria are:

$$\phi(0) < \phi\left(\frac{1}{M}\right)$$

$$\phi'\left(\frac{1}{M}\right) < 0$$

We find that in order to have stable symmetric fixed point in this model, $\phi(A_i)$ has to be non-monotonic: it rises at first due to the first condition, and then drops due to the second condition.

To derive explicit conditions, we next assume that $\phi$is parabolic (Fig. 2b) of the form:

$$\phi(A_i) = -\left(\frac{A_T}{M_1} - A_i\right)\left(\frac{A_T}{M_2} - A_i\right)$$

Applying our stability criteria, we find:

$$\frac{A_T M_{max}}{2} < M < A_T M_{max}$$

with $M_{max} = \frac{2M_1 M_2}{M_1 + M_2}$

The growth rate of follicles in the symmetric solution is:

$$\frac{dx_i}{dt}\left(\frac{x_T}{M}\right) = -\frac{1}{M}\cdot\left(\frac{1}{M_1} - \frac{A_t}{M}\right)\left(\frac{1}{M_2} - \frac{A_t}{M}\right) = v_M$$

The follicles grow linearly with time with velocity v_M if $M_1 < M < M_2$, otherwise they shrink linearly. In order to achieve ovulation, the follicles must grow at a positive velocity, which requires:

$$A_T M_1 < M < A_T M_2$$

In order to have a stable growing solution, we combine the stability criteria with the growth criteria:

$$A_T M_1 < M < A_T M_{max}$$

Notice $M_{max} < 2M_1$, thus we get a theoretical limit for the ovulation number (Fig 3C)- it cannot be larger than two times the minimal ovulation numer. This raises the question of dizygotic twins and triplets in women, which show an ovulation number two or three times larger than the minimal ovulation number of 1. We suggest that twins and triplets represent ovulation events which are not fixed points but rather occur close to an unstable solution.

We now consider the second family of fixed points, the semi-symmetric solutions. We show that they are all unstable for our choice of parabolic ϕ (or more generally a unimodal ϕ). The follicles can be divided to two groups: a group of A follicles with relative size u_A in the rising part of ϕ, and a group of B follicles with relative size u_B in the declining part of ϕ

$$\phi(u_A) = \phi(u_B) = C = \sum_{j=1}^{N} u_j \phi(u_j)$$

$$\sum_{i}^{M} u_i = Au_A + Bu_B = 1$$

For the parabolic ϕ we find:

$$u_A = \frac{1}{A-B} - \frac{B}{A-B}\frac{M_1+M_2}{M_1M_2}$$

$$u_B = -\frac{1}{A-B} + \frac{A}{A-B}\frac{M_1+M_2}{M_1M_2}$$

We can derive the Jacobian for these solutions as well, however we can intuitively see these solutions are unstable because group A resides in the rising part of $\phi$and a small deviation from the solution will send the follicles away from u_A(Fig. S5)

Notice that A, B must be natural numbers, thus these unstable solutions may not always be possible, depending on the choice of M_1, M_2, M.

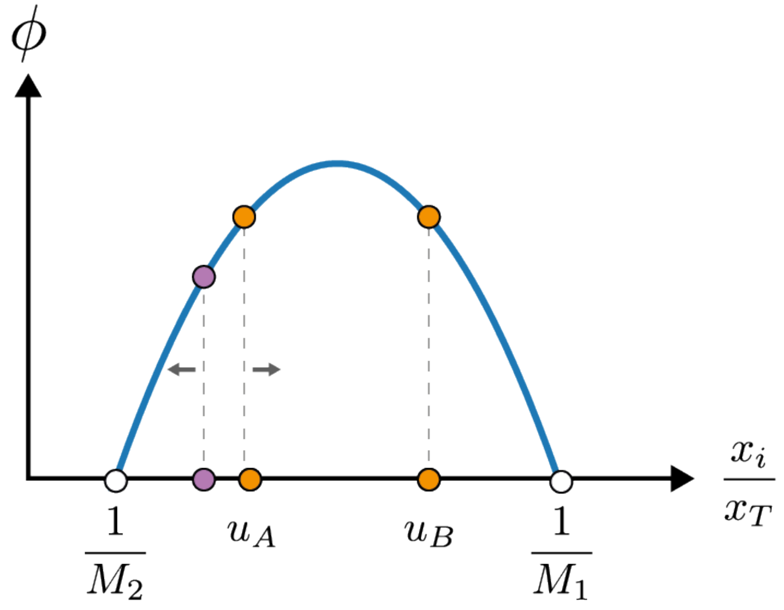

Figure S5. The semi-symmetric solution is unstable for our choice of ϕ. In the semi-symmetric solutions for ϕ which is parabolic, there are two groups of follicles, one with A follicles and the other with B follicles, with relative sizes u_A, u_B accordingly, such that $\phi(u_A) = \phi(u_B)$. If one of the follicles with relative size $u_A$gets a little bit smaller than the others, it will grow slower than the others and its relative size will continue to shrink further away from u_A, such that the

deviation is amplified. Alternatively, if the follicle is a little bit larger than the others with u_A, it will grow faster than the others and its relative size will continue to grow. Therefore, the semi-symmetric solution is unstable.

The general behavior of the model with our choice of ϕ can be easily understood by examining the case of three growing follicles. Looking at relative-size space, the constraint $\sum_{i=1}^{3} u_i = 1$ defines an equilateral triangular plane (blue plane in Figure S6). Each vertex of the triangle represents a solution where the relative size of one of the follicles equals 1, and the relative size of the other two is zero; each midpoint of an edge represents a solution where two follicles have a relative size of half and the third follicle has a relative size of zero, and the center of the triangle represents a solution where the relative size of each of the follicles is $\frac{1}{3}$. To understand stability, it is sufficient to examine the triangle. In Figure S7 we plot the phase portrait of this case for different parameter choices. The trajectories clearly show that $M = 1$, $M = 2$ or $M = 3$ can be stable (black circles are stable fixed points), and also a case where both $M = 2$ and $M = 3$ are stable is possible, and that there exist unstable fixed points (white circles are sources and blue circles are saddles).

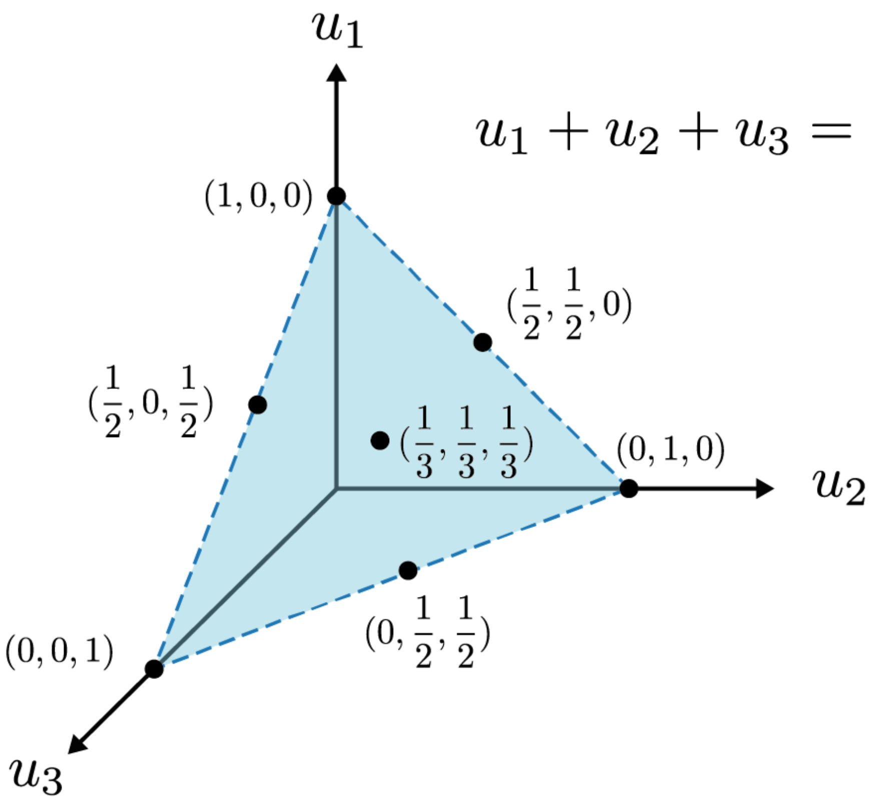

Figure S6. A system of three growing follicles defines an equilateral triangular plane in the relative-size space. Relative sizes of three follicles u_1, u_2, u_3 should satisfy $u_1 + u_2 + u_3 = 1$ and $u_1, u_2, u_3 > 0$, hence they define an equilateral triangular plane in the relative-size space. The plane is marked in blue, and the black dots represent the symmetric solution fixed points.

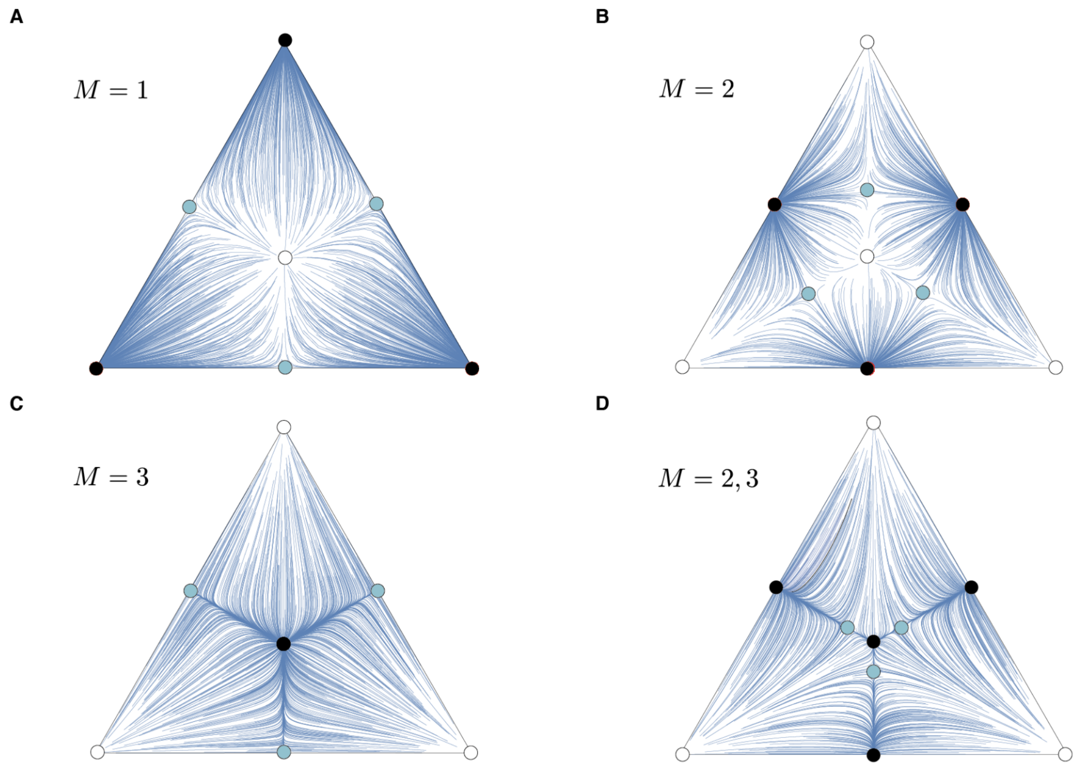

Figure S7. Simulations of a system of three growing follicles show convergence to stable fixed points (black dots) for symmetric solutions and existence of unstable fixed points (white dots - sources, cyan dots - saddles) of symmetric solutions and semi-symmetric solutions in the relative-size space. (A) Simulations of our model with three follicles, with $M_1 = 0.5, M_2 = 10$, show stable fixed-points in the relative-size space for the symmetric solutions of $M = 1$, unstable fixed point for the symmetric solutions of $M = 2,3$ (B) Simulations of our model with three follicles, with$M_1 = 1.5, M_2 = 10$show stable fixed-points in the relative-size space for the symmetric solutions of $M = 2$, unstable fixed point for the symmetric solutions of $M = 1,3$ (C) Simulations of our model with three follicles, with $M_1 = 2.5, M_2 = 10$, show a stable fixed-point in the relative-size space for the symmetric solution of $M = 3$, unstable fixed point for the symmetric solutions of $M = 2,3$(D) Simulations of our model with three follicles, with$M_1 = 1.75, M_2 = 20$show stable fixed-points in the relative-size space for the symmetric solutions of $M = 2,3$, and unstable fixed points for the symmetric solutions of $M = 1$.

S3.3. The model shows PCOS-like behavior at high exogenous androgen

We next consider the effect of exogenous androgen. This could be androgen produced by the adrenal glands during hyperactivity, consumed androgen steroids, or androgen produced in the ovary independently of the control of LH. For example, it seems in some women with PCOS insulins resistance increases androgen production in the ovary, and treatment with metformin may improve both insulin resistance and androgen levels, and regain fertility[7]–[10].

Exogenous androgen A_{ex} can be modelled as an additive term to the local androgen $\frac{x_i}{x_T}$, as follows:

$$\frac{dx_i}{dt} = -\frac{x_i}{x_T} \cdot \left(1 - M_1\left(\frac{x_i}{x_T} + A_{ex}\right)\right)\left(1 - M_2\left(\frac{x_i}{x_T} + A_{ex}\right)\right)$$

In the symmetric solution with$M$equal follicles we find:

$$\frac{dx_i}{dt} = -\frac{1}{M} \cdot \left(1 - \left(\frac{M_1}{M} + M_1 A_{ex}\right)\right)\left(1 - \left(\frac{M_2}{M} + M_1 A_{ex}\right)\right)$$

If excess androgen is high, $A_{ex} > \frac{1}{M_1} - \frac{1}{M}$, the follicles have a negative velocity and shrink linearly with time. For lower excess androgen, $A_{ex} < \frac{1}{M_1} - \frac{1}{M}$ the follicles grow with a constant positive velocity. Thus, A_{ex} greater than a threshold $\frac{1}{M_1} - \frac{1}{M}$ causes the follicles to shrink.

Adding A_{ex} effectively shifts the parabola to the left, so that the fixed points are now at:

$$a_1 = \frac{1}{M_1} - A_{ex}$$

$$a_2 = \frac{1}{M_2} - A_{ex}$$

Meaning we now have an effective $\widetilde{M}_1 = \frac{M_1}{1 - M_1 A_{ex}}$, $\widetilde{M}_2 = \frac{M_2}{1 - M_2 A_{ex}}$, where both the minimal and maximal number of possible ovulations is larger than without external androgen. If $M < \widetilde{M}_1$ all follicles shrink, which we believe may be a reasonable model for PCOS. Our model doesn't capture the initial follicular growth seen in longitudinal ultrasound imaging of PCOS patients. However, note that we interpret x_i as the functional estradiol secreting mass (granulosa cells) of the follicle. In PCOS patients, the granulosa cells layer becomes thin during follicular growth[11], while the antral cavity fills with fluid, so that measurements of diameter in PCOS may not be simply related to x_i in the model.

S4. Polynomial dependence of follicle mass on time

We note that a slightly more complex model can be formed in which follicle mass rises polynomially with time, instead of linearly. This can take into account the relation between follicle mass and follicle diameter. Since theca and granulosa cells are mainly on the spherical edge of the follicle, in a layer that changes thickness over time, experiments show that their number or mass scales as diameter to a power between one and two [12] (Fig S8).

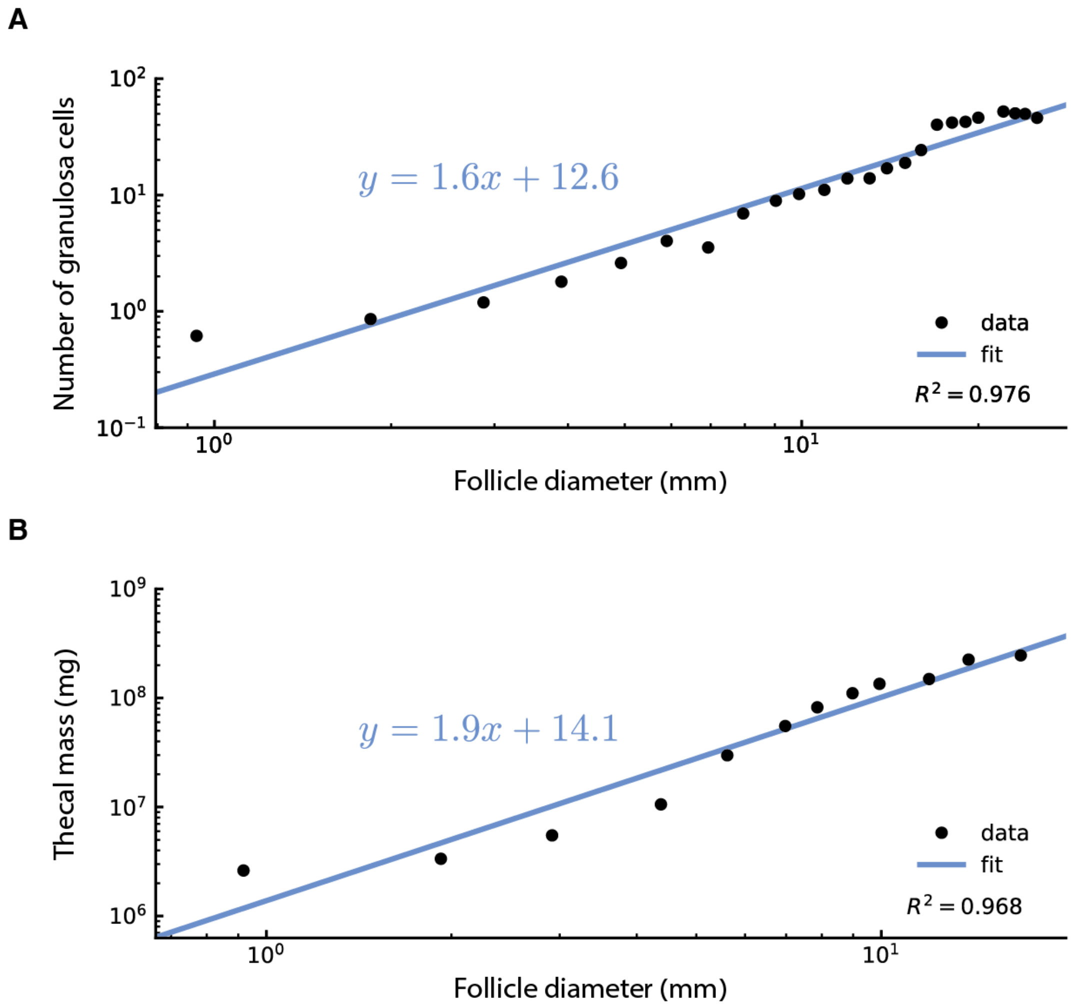

Figure S8. Number of granulosa cells and theca mass grow as follicle diameter to a power between 1 and 2 (A) Number of granulosa cell scales as diameter of the follicle with a power of 1.6 ± 0.1, adapted from [12].(B) Mass of theca cells scales as diameter of the follicle with a power of 1.9 ± 0.2, adapted from [12].

Additionally, FSH dependence of estradiol need not be exactly $FSH \sim \frac{1}{E}$ as assumed in the main text, but rather more like $FSH \sim \frac{1}{E^q}$, with experiments suggesting q less than one [3] (Fig S2). Thus

$$\frac{dx_i}{dt} = \frac{x_i}{{x_T}^q}\phi(\frac{x_i}{x_T})$$

In the symmetric solution, where$x_i = \frac{x_T}{M}$,

$$\frac{dx_T}{dt} = {x_T}^{1-q}\phi(\frac{1}{M})$$

The solution is polynomial growth with time of each $x_i$and of total mass

$$x_T \sim t^{\frac{1}{q}}$$

Thus if $q = 0.5$, we obtain that follicle mass grows as t^2, and if $q = 1$ as assumed in the main text, follicles grow linearly as t. Such a model allows the diameter of the dominant follicle(s) to grow linearly with time and their mass to grow polynomially with time.

References

[1] A. Schneyer *et al.*, “Dynamic Changes in the Intrafollicular Inhibin/Activin/Follistatin Axis during Human Follicular Development: Relationship to Circulating Hormone Concentrations 1,” *The Journal of clinical endocrinology and metabolism*, vol. 85, pp. 3319–30, Oct. 2000, doi: 10.1210/jcem.85.9.6767.

[2] A. R. Baerwald, G. P. Adams, and R. A. Pierson, “A new model for ovarian follicular development during the human menstrual cycle,” *Fertility and Sterility*, vol. 80, no. 1, pp. 116–122, Jul. 2003, doi: 10.1016/S0015-0282(03)00544-2.

[3] R. I. Mclachlan, N. L. Cohen, K. D. Dahl, W. J. Bremner, and M. R. Souls, “Serum inhibin levels during the periovulatory interval in normal women: relationships with sex steroids and gonadotrophin levels,” *Clinical Endocrinology*, 1990, doi: 10.1111/j.1365-2265.1990.tb03748.x.

[4] G. E. Abraham, “Ovarian and Adrenal Contribution to Peripheral Androgens During the Menstrual Cycle,” *The Journal of Clinical Endocrinology & Metabolism*, vol. 39, no. 2, pp. 340–346, Aug. 1974, doi: 10.1210/jcem-39-2-340.

[5] “Hormone seasonality in medical records suggests circannual endocrine circuits | PNAS.” https://www-pnas-org.ezproxy.weizmann.ac.il/content/118/7/e2003926118.short (accessed Jul. 13, 2021).

[6] H. M. Lacker, “Regulation of ovulation number in mammals. A follicle interaction law that controls maturation,” *Biophysical Journal*, vol. 35, no. 2, pp. 433–454, 1981, doi: 10.1016/S0006-3495(81)84800-X.

[7] R. J. Chang and D. A. Dumesic, “Chapter 21 - Polycystic Ovary Syndrome and Hyperandrogenic States,” in *Yen and Jaffe’s Reproductive Endocrinology (Eighth Edition)*, J. F. Strauss and R. L. Barbieri, Eds. Philadelphia: Elsevier, 2019, pp. 520-555.e13. doi: 10.1016/B978-0-323-47912-7.00021-4.

[8] E. M. Velazquez, S. Mendoza, T. Hamer, F. Sosa, and C. J. Glueck, “Metformin therapy in polycystic ovary syndrome reduces hyperinsulinemia, insulin resistance,

hyperandrogenemia, and systolic blood pressure, while facilitating normal menses and pregnancy," *Metabolism*, vol. 43, no. 5, pp. 647–654, May 1994, doi: 10.1016/0026-0495(94)90209-7.

[9] A. Dunaif, "Insulin resistance and the polycystic ovary syndrome: mechanism and implications for pathogenesis," *Endocr Rev*, vol. 18, no. 6, pp. 774–800, Dec. 1997, doi: 10.1210/edrv.18.6.0318.

[10] P. Moghetti and F. Tosi, "Insulin resistance and PCOS: chicken or egg?," *J Endocrinol Invest*, vol. 44, no. 2, pp. 233–244, Feb. 2021, doi: 10.1007/s40618-020-01351-0.

[11] R. J. Chang and D. A. Dumesic, *Polycystic Ovary Syndrome and Hyperandrogenic States*, no. 2. Elsevier Inc., 2019. doi: 10.1016/B978-0-323-47912-7.00021-4.

[12] K. P. Mc Natty, "Hormonal correlates of follicular development in the human ovary," *Australian Journal of Biological Sciences*, vol. 34, no. 3, pp. 249–268, 1981, doi: 10.1071/BI9810249.
